# Nandrolone decanoate impairs gastrointestinal motility and duodenal morphometry in moderately exercised rats

**DOI:** 10.1590/1414-431X2024e13452

**Published:** 2024-07-01

**Authors:** A.T. Hauschildt, L.A. Gama, G.T. Volpato, L.A. Corá, A.A.V. Silva, M.O. Belém, P.J.C. Magalhães, A.A. Santos, M.F. Américo

**Affiliations:** 1 Instituto de Ciências Biológicas e da Saúde, Universidade Federal de Mato Grosso, Barra do Garças, MT, Brasil; 2 Centro de Ciências Integradoras, Universidade Estadual de Ciências da Saúde de Alagoas, Maceió, AL, Brasil; 3 Departamento de Fisiologia e Farmacologia, Universidade Federal do Ceará, Fortaleza, CE, Brasil

**Keywords:** Anabolic androgenic steroids, Gastrointestinal motility, Gastrointestinal transit, Swimming

## Abstract

The misuse of anabolic androgenic steroid associated or not with physical workouts disrupts gastrointestinal (GI) function homeostasis. Our goal was to investigate the effects of nandrolone decanoate (ND) and moderate swimming on the GI transit of solid meals, GI motor contractility, and intestinal histology in rats. Male Wistar rats were allocated to four groups that received intramuscular injections of ND (5.0 mg/kg) or vehicle (60.0 µL) and were submitted or not to swimming sessions (60 min, 5% body weight overload) for 4 weeks. Gastric emptying, intestinal transit, *in vitro* GI contractility, intestinal morphometry, and duodenal mucosal mast cells were evaluated in all experimental groups. ND treatment accelerated gastric emptying, slowed small intestine transit time, enhanced gastric carbachol-mediated reactivity, decreased crypt depth and villus height, reduced mucosal thickness, and increased the circular and longitudinal muscle layer thickness of the duodenum in sedentary rats. Moderate exercise accelerated intestinal transit time and reduced submucosa thickness. In vehicle-treated animals, a strong negative correlation was found between intestinal transit and mucosal mast cells, which was reversed by ND treatment. Combining ND treatment and swimming accelerated gastric emptying, increased duodenal cholinergic reactivity, inhibited the sodium nitroprusside relaxing response, increased the number of duodenal mast cells, decreased villus height, and increased the thickness of all muscle layers. ND changed the morphological and functional properties of the GI tract over time, with intense dysmotility, especially in sedentary animals, but moderate exercise seemed to have played a compensatory role in these harmful effects in the gut.

## Introduction

Anabolic androgenic steroids (AAS) are synthetic molecules chemically similar to testosterone that were originally synthesized for treating hypogonadism (1). Nandrolone decanoate (ND) is one of the most popular injectable drugs prescribed as anabolic therapy for several clinical conditions including burns, radiation therapy, surgery, trauma, anemia, chronic kidney disease, and osteoporosis in postmenopausal women, among others (2). However, over the years, ND has been commonly misused in higher than therapeutic doses for improving physical performance and body composition (3). Supratherapeutic doses can lead to a wide range of side effects in several organs and systems, both in animals and humans (2,4). ND has shown deleterious effects on the nervous system, impairing central neurotransmission via GABAA, serotonin, and dopamine receptors, decreasing cell proliferation and neurogenesis, and impairing cardiac and vascular autonomic regulation (1,5,6).

The gastrointestinal (GI) tract is a potential target of endogenous AAS (7-9) and thus susceptible to the action of testosterone derivatives - either by direct interaction or aromatization into estradiol (2). AAS misuse can disturb gut physiology by eliciting malabsorption, nausea, vomiting, diarrhea, GI irritation, and abdominal pain (10). Furthermore, physical activity has been prescribed for the management of gut dysmotility (11), but the effects of physical exercise on GI motor behavior are controversial (12-14) and influenced by differences in experimental conditions and protocols (13).

Gut motility is closely controlled by a complex neuroendocrine network, including mucosal mast cells (15). Mast cells are widely distributed in the gut and influence blood flow, smooth muscle contractions, peristalsis, mucosal secretion, and immune response (16). Several stimuli activate mast cells, which release agents that can disrupt the integrity of the gut epithelial barrier and its permeability (16). Moreover, the direct interaction between mast cells and enteric nervous system cells may also alter the motility patterns of the gut (15).

The relationship between AAS misuse, physical exercise, and GI motility, including mast cell activation, remains unclear. Studies using animal models provide valuable information about the side effects of AAS misuse in several conditions. Additionally, animal models are also useful in addressing issues regarding pathophysiological mechanisms, generating solid experimental evidence with a potential contribution to translational research (17). Thus, the present study investigated the combined effects of ND and moderate swimming workout on the gut transit of a solid meal, GI motor contractility, intestinal morphometry, and mucosal mast cells in rats.

## Material and Methods

### Ethical approval

All of the experimental procedures were conducted according to the National Institutes of Health Guide for the Care and Use of Laboratory Animals, following the Animal Research: Reporting of *In Vivo* Experiments protocol, and after approval by the Ethics Committee on Animal Research of the Federal University of Mato Grosso (protocol No. 23108.049862/13-3).

### Animals and experimental design

Male Wistar rats (weighing initially 250-300 g, n=28 animals) were randomly assigned to one of four groups (n=7 animals/group) treated with vehicle (peanut oil) or ND: sedentary vehicle (SV; sedentary animals treated with vehicle), exercise vehicle (EV; animals trained to swim and treated with vehicle), sedentary ND (SN; sedentary animals treated with ND), and exercise ND (EN; animals trained to swim and treated with ND). The animals were housed in polycarbonate cages with wire lids and hardwood chips for bedding and were maintained under controlled conditions of temperature (22±1°C), humidity (50±10%), and a 12-h light/dark cycle with free access to chow and water. Body weight was measured daily at 7:00 am and weekly weight gain was determined as the difference between the weight on the last and first day of the week. These measurements were used to assess the correlation between weight loss and ND treatment.

### Drug and vehicle treatment protocols

ND (Deca-Durabolin, Schering-Plough, Brazil) at 5.0 mg/kg or peanut oil (60.0 µL), according to the experimental group, was administered by intramuscular injection 5 days per week for 4 weeks. The injections were given alternately in the left and right hind leg. ND dose was based on previous studies in rodents (18,19).

### Exercise protocol

The exercise program was adapted from de Araújo et al. (20). Our study adopted a swimming protocol with a load of 5% of body weight. The intensity of this protocol is considered moderate. However, to confirm this information, blood lactate was measured. Two weeks before the tests, rats were exposed to water for 10 consecutive days. Firstly, they were placed in tanks (80×150 cm) filled to a maximum depth of 10 cm of water at 31±1°C for 10 min daily for 5 days. For the next 5 days, the exposure time increased gradually by 10 min per day until a maximum of 60 min. At this stage, two animals from the trained group were simultaneously placed in the same tank (∼100 cm water depth) and the sedentary animals remained in the shallow water (10 cm). Adaptation periods were necessary to reduce physiological changes due to stress since forced swimming was not applied (20,21).

The exercise protocol consisted of moderate-intensity swimming. Trained rats were placed individually for 60 min in the same tanks filled with water (31±1°C) to a depth of 100 cm, and with a 5% body weight load attached to their tail. Swimming sessions were performed 5 days/week for 4 weeks in the morning and immediately after ND or vehicle administration. Sedentary animals remained in shallow water for the same time interval without the weight load (21). Trained animals were kept in the swimming status throughout the training session under continuous supervision by the authors, preventing them from reaching the bottom or hanging on the side of the tank (20,22). Animals that showed persistent immobility in the tank were excluded from the study (2 in the EV group and 1 in the EN group), thus only the active ones remained in the study (n=7).

### Body composition and blood lactate assessment

Bioimpedance spectroscopy (Vet BIS1, ImpediMed, Australia) was performed after short-acting anesthesia with 50 mg/kg ketamine (*ip,* Cetamin, Syntec, Brazil) plus 5 mg/kg xylazine hydrochloride solution (*ip,* Xilazin, Syntec) on days 1, 14, and 28. The following parameters were used to calculate body composition: total body water (TBW), intracellular fluid (ICF), extracellular fluid (ECF), fat-free mass (FFM), fat mass (FM), and body mass index (BMI). Blood samples (25 μL) were collected from the tail vein immediately after the training sessions on day 26 of treatment to determine blood lactate concentrations using the Accutrend test-strip system (Accutrend Plus, Roche Diagnostics, Germany).

### Gastrointestinal transit measurements

Gut motility was assessed by alternating current biosusceptometry (ACB) using an ACB magnetic sensor (Br4-Science, Brazil). This technique was previously applied to evaluate GI motility in rodents (23,24). Briefly, the ACB sensor detects magnetic signals generated from a magnetic test meal, whose signal intensity depends on the amount of magnetic material and the distance between the sensor and the magnetic sample (23). Ferrite powder (MgZnFe_2_O_3_, Imag, Brazil) was previously incorporated into the rats' laboratory chow and served as the nonabsorbable magnetic marker. Detailed technical information has been reported elsewhere (24).

After fasting overnight, the animals ingested the magnetically marked chow (0.4 g ferrite powder and 1.6 g laboratory chow). Afterward, the ACB sensor was gently placed on the animals' abdominal surface to monitor the displacement of the magnetically marked chow from the gastric to the cecum projection. These measurements were performed in awake rats at regular 15-min intervals for at least 6 h (24) and repeated in all animals on days 1, 14, and 28 after treatments.

Gastric emptying (GE) and cecum arrival (CA) were determined as differences in the intensity of the magnetic signals from the stomach to the cecum. Data were analyzed in Origin software (OriginLab Northampton, USA), and statistical moments (25) were applied to determine the mean GE time (defined as the time *t* [in minutes] at which a mean amount of the magnetic meal was emptied from the stomach, calculated as the area under the emptying curve) and the mean CA time (defined as the time *t* [in minutes] at which an increase in the mean amount of magnetic meal arrived in the cecum, calculated as the area between the cecum arrival curve and maximal cumulative values). Mean small intestine transit time was quantified as the difference between CA time and GE time (24,25).

### Tissue sample collection

At the end of the 28-day protocol, the animals were euthanized by an intraperitoneal overdose of 240 mg/kg ketamine (Cetamin, Syntec) plus 45 mg/kg xylazine hydrochloride solution (Xilazin, Syntec).

### *In vitro* gastrointestinal contractions

Immediately after euthanasia, isolated strips of the gastric fundus, gastric antrum, and duodenum were collected. The stomach was opened along the lesser curvature, and 1 cm-long strips were cut from the antrum and fundic region. The duodenum was cut into 1 cm-long longitudinal segments. Tissues were maintained in physiological solution (modified Tyrode solution: 136 mM NaCl, 5 mM KCl, 0.98 mM MgCl_2_, 2 mM CaCl_2_, 0.36 mM NaH_2_PO_4_, 11.9 mM NaHCO_3_, and 5.5 mM glucose) at room temperature and rapidly transferred to bath chambers for contractile recordings. For this purpose, tissues were mounted while considering the longitudinal orientation of the smooth muscle layers. Experimental bath chambers (5 mL) were used to simulate physiological conditions and maintain tissue viability. Each bath chamber was filled with Tyrode solution and continuously aerated with a mixture of 5% CO_2_ in 95% O_2_, maintained at 37°C and pH 7.4. Tissues were tied with inextensible cotton thread, with one extremity attached to a fixed pin in the bath chamber and the other extremity attached to a force transducer (MLT0201, AD Instruments, Australia) coupled to a data acquisition system (PowerLab 8/30, AD Instruments). A basal tension of 1 g was applied to the tissues. They were initially maintained under these conditions for 20-30 min for equilibration, followed by the renewal of Tyrode solution and tension adjustments at 10 min intervals (26,27).

Tissue viability was evaluated by an initial addition of 60 mM KCl. Segments without spontaneous activity were discarded. The strips were subjected to increasing concentrations of KCl (10-140 mM), carbachol (CCh; 0.01-30 μM), isoproterenol (ISO; 0.1-30 μM), and sodium nitroprusside (SNP; 0.1-30 μM) to generate concentration-effect curves for contractile or relaxant agents, which were obtained from the asymptotic value recorded at each concentration. Relaxing responses were measured after initial stimulation with 10 μM CCh. *In vitro* GI contractile activity data are reported as a percentage of the maximal tension attained during the initial 60 mM KCl challenge (27).

### Histological analyses

Tissue samples of the duodenum were fixed in Methacarn (60% methanol, 30% chloroform, and 10% glacial acetic acid), dehydrated in an ascending series of alcohol (70, 90, 96, and 100%), cleared in xylene, and embedded in paraffin. Semiserial 4-μm sections were cut with a microtome (HM-355S Automatic Microtomes, Thermo Scientific, Germany) and stained with 0.5% toluidine blue or hematoxylin and eosin (HE). Toluidine blue-stained sections were used to identify mast cells in the mucosa. Mast cell granules exhibit metachromatic staining after the uptake of toluidine blue dye. Sections of the duodenum were randomly selected from each rat. Twenty well-oriented villus-crypt units (VCU) were examined per animal and the number of mucosal mast cells per VCU was recorded. Hematoxylin and eosin staining were used for the morphometric assessment of the total intestinal wall, villus height, crypt depth, and thickness of the mucosa, submucosa, and external muscle (circular and longitudinal layers) (28). Images were captured with an optical microscope (Zeiss, Germany) coupled to a high-resolution camera (Moticam, Hong Kong) and analyzed using ImagePro software (Media Cybernetics, USA). Eight histological sections were photographed per animal, and 10 different measurements were obtained for each parameter. The number of mucosal mast cells and morphometry were assessed in a blinded manner to avoid bias.

### Statistical analyses

Statistical analysis was performed using the Prism software (GraphPad, USA). The Shapiro-Wilk normality test was initially used to evaluate data distribution. One-way ANOVA followed by the Tukey *post hoc* test was applied to the normal distribution analyses, i.e. gastrointestinal transit measurements, mast cell quantification, body composition, and blood lactate. Kruskal-Wallis test followed by Dunn's multiple-comparison *post hoc* test were used when data showed non-normal distribution, i.e. *in vitro* reactivity assay and duodenal morphometry. Cohen's d values were calculated to determine the effect sizes using means, standard deviations, and sample size. The effect-size measure was applied to verify the eventual difference between two variables and was defined as small, medium, or large if the effect size was 0.2, 0.5, or 0.8, respectively (29). The Pearson's correlation coefficient (R) was determined to assess eventual relationships between intestinal variables. Only coefficients above 0.80 were considered biologically relevant. Differences were considered significant at P<0.05. All data are reported as means±SE or median and interquartile range (25-75%).

## Results

### Nandrolone decanoate impaired gastrointestinal transit over time

**Figure 1:**
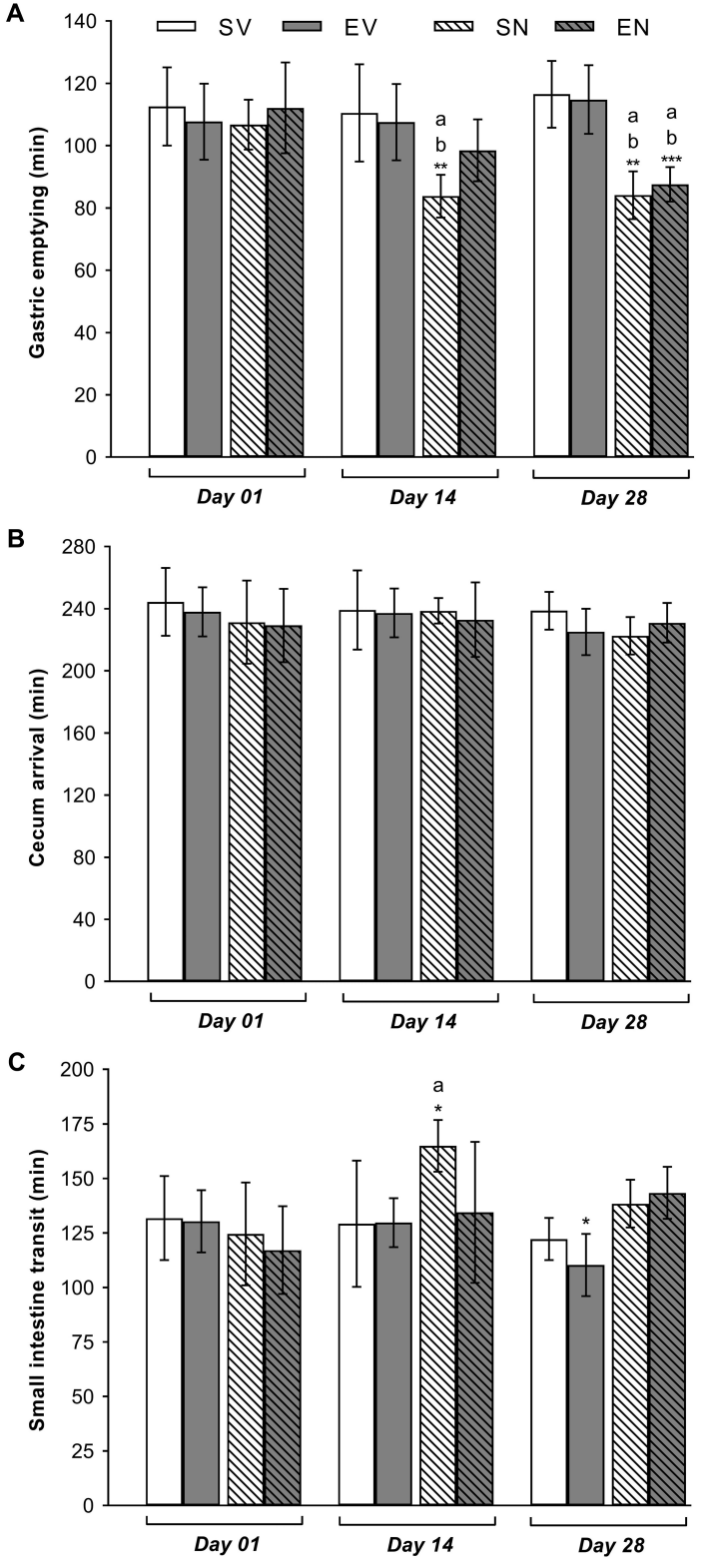
Mean values of (**A**) gastric emptying, (**B**) cecum arrival, and (**C**) small intestine transit time of a solid test meal in sedentary vehicle (SV), exercise vehicle (EV), sedentary 5 mg/kg nandrolone (SN), and exercise 5 mg/kg nandrolone (EN) rats on days 1, 14, and 28 of treatment. Data are reported as means±SE from n=7 animals/group. ^a^P<0.050 *vs* SV; ^b^P<0.010 *vs* EV; *P<0.010 *vs* EV on day 1; **P<0.010 *vs* SN on day 1; ***P<0.001 *vs* EN on day (ANOVA followed by Tukey's *post hoc* test).

ND treatment accelerated the GE rate in awake rats (Figure 1A). This effect was observed over time, regardless of the combination with swimming workouts. Compared with the respective vehicle-treated group (112.13±5.65 min), a faster GE rate was observed in the SN group on day 14 of treatment (83.76±3.08 min), which persisted until day 28 of ND treatment (85.48 ± 3.67 min; Cohen's d=22.18). Compared with the respective GE values of sedentary animals (118.36±3.98 min), the EN group exhibited a significantly higher GE rate (87.12±2.97 min) at the end of the swimming protocol. As shown in Figure 1B, there were no significant changes in cecum arrival time among all groups. The swimming protocol accelerated the small intestine transit time in the EV group on day 28 compared with the respective basal values on day 1 (134.76±3.59 min *vs* 107.20±7.41 min) (Figure 1C). Moreover, ND treatment delayed small intestine transit time on day 14 (165.90±5.19 min *vs* 128.61±9.49 min for the SV group) (Figure 1C).

### Changes in gastrointestinal contractility

**Figure 2:**
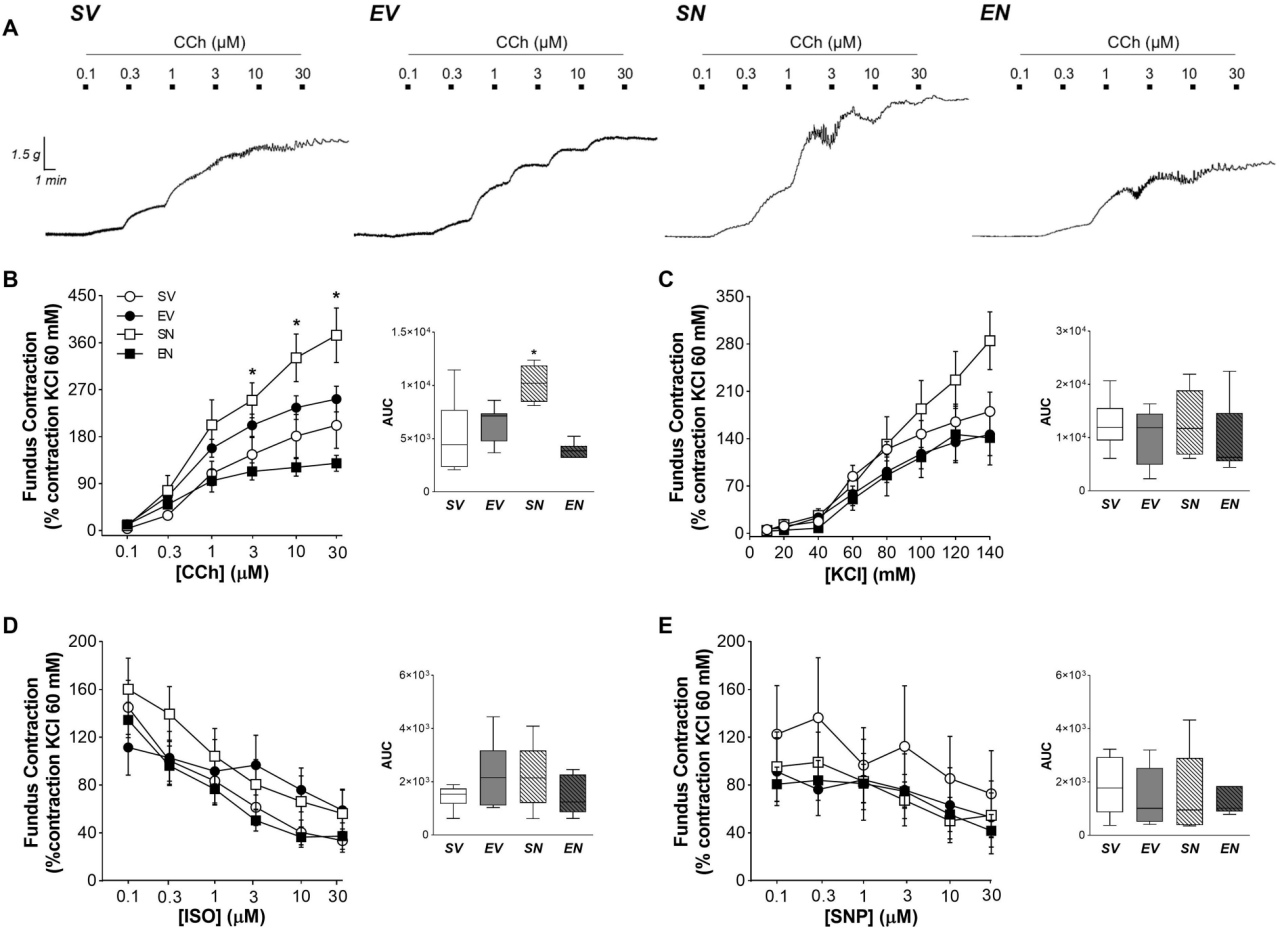
**A**, Representative curves of fundic strips exposed to increasing concentrations of carbachol (CCh). Contractile response to (**B**) CCh (0.01-30 μM), (**C**) KCl (10-140 mM), (**D**) isoproterenol (ISO; 0.1-30 μM), and (**E**) sodium nitroprusside (SNP; 0.1-30 μM) in isolated strips of the gastric fundus of sedentary vehicle (SV), exercise vehicle (EV), sedentary 5 mg/kg nandrolone (SN), and exercise 5 mg/kg nandrolone (EN) rats after the 28-day protocol. AUC: area under the curve. Data are reported as median and interquartile range (25-75%) from n=7 animals/group. *P<0.050 *vs* SV (Kruskal-Wallis test followed by Dunn's *post hoc* test).

**Figure 3 f03:**
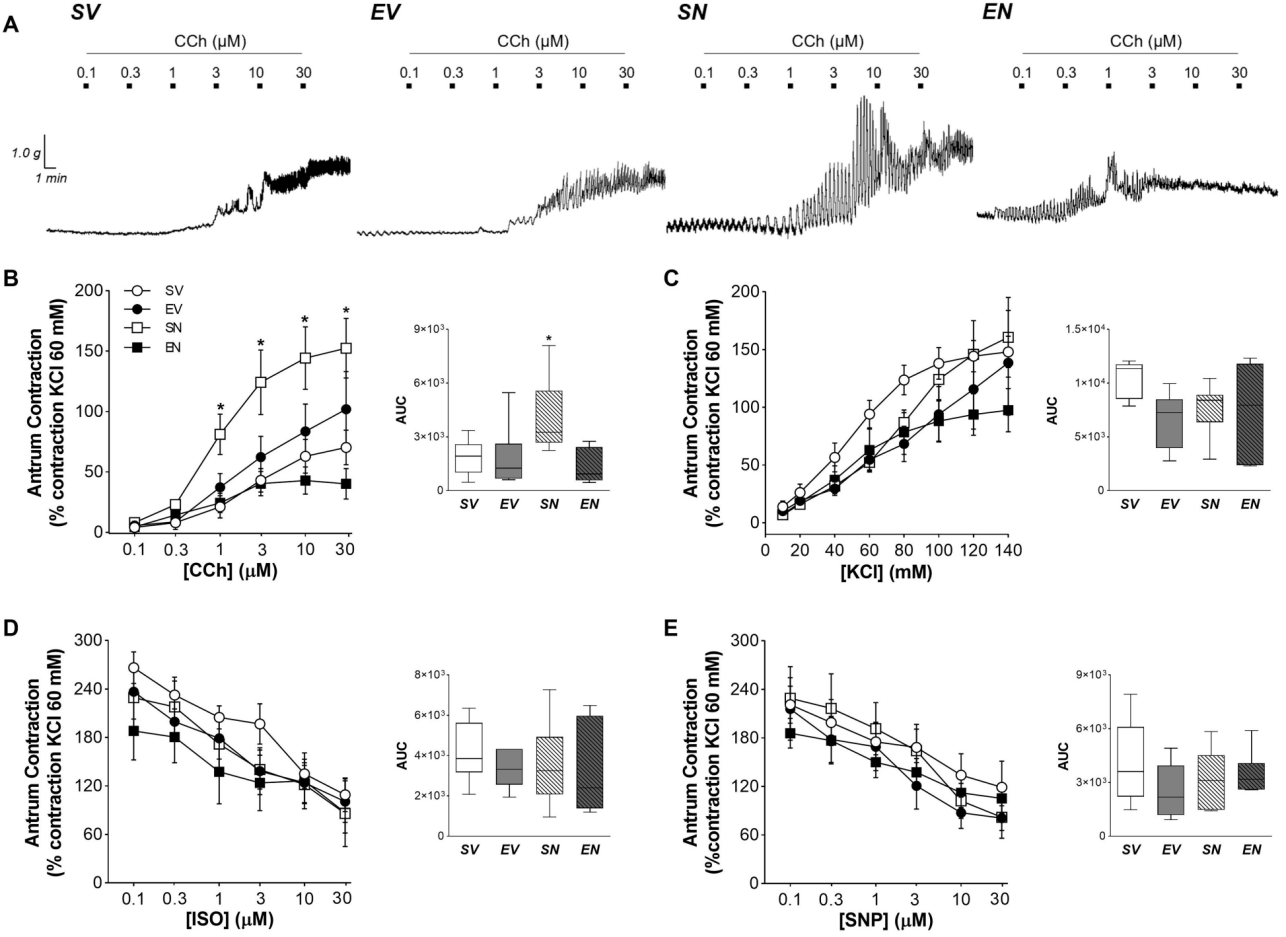
**A**, Representative curves of antrum strips exposed to increasing concentrations of carbachol (CCh). Contractile response to (**B**) CCh (0.01-30 μM), (**C**) KCl (10-140 mM), (**D**) isoproterenol (ISO; 0.1-30 μM), and (**E**) sodium nitroprusside (SNP; 0.1-30 μM) in isolated strips of the gastric antrum of sedentary vehicle (SV), exercise vehicle (EV), sedentary 5 mg/kg nandrolone (SN), and exercise 5 mg/kg nandrolone (EN) rats after the 28-day protocol. AUC: area under the curve. Data are reported as median and interquartile range (25-75%) from n=7 animals/group. *P<0.010 *vs* SV (Kruskal-Wallis test followed by Dunn's *post hoc* test).

**Figure 4:**
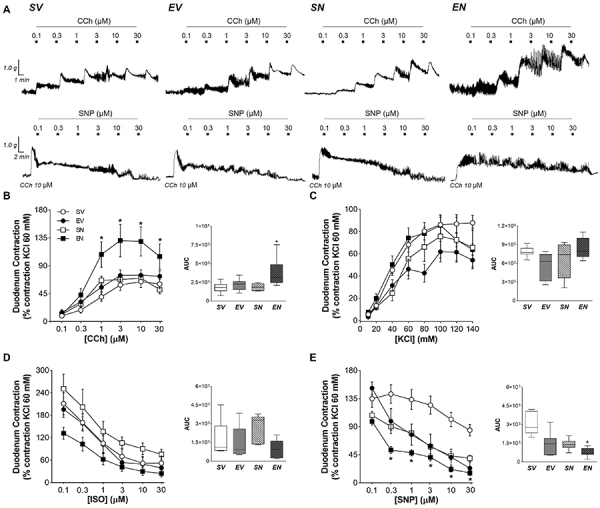
**A**, Representative curves of duodenal strips exposed to increasing concentrations of CCh and SNP. Contractile response to (**B**) CCh (0.01-30 μM), (**C**) KCl (10-140 mM), (**D**) isoproterenol (ISO; 0.1-30 μM), and (**E**) sodium nitroprusside (SNP; 0.1-30 μM) in isolated strips of the duodenum of sedentary vehicle (SV), exercise vehicle (EV), sedentary 5 mg/kg nandrolone (SN), and exercise 5 mg/kg nandrolone (EN) rats after the 28-day protocol. AUC: area under the curve. Data are reported as median and interquartile range (25-75%) from n=7 animals/group. *P<0.010 *vs* SV (Kruskal-Wallis test followed by Dunn's multiple-comparison *post hoc* test).

The concentration-effect curves of the contractile and relaxant agents in the *in vitro* preparations of isolated strips from gastrointestinal segments are shown in Figures 2-4. In sedentary animals, both gastric segments exhibited a significant increase in carbachol-mediated contractility after ND treatment (fundus area under the curve (AUC): 10210 [8462; 11868] *vs* 4439 [2314; 7694] for SV group, P<0.05; antrum AUC: 3255 [2677; 5571] *vs* 1927 [1021; 2599] for SV group, P<0.01), without interfering with the depolarizing stimulus (i.e. KCl crescent concentrations) or inhibitory responses to ISO and SNP (Figures 2 and 3). The exercise protocol combined with ND treatment markedly reduced the concentration-response curves of the relaxing agent SNP (AUC: 645 [581; 1099] *vs* 2753 [2322; 4071] for SV group, P<0.001) but enhanced cholinergic transmission in the duodenum (AUC: 3136 [2439; 4882] *vs* 1795 [1344; 2237] for SV group, P<0.05) (Figure 4).

### Histological alterations in the duodenum

**Figure 5:**
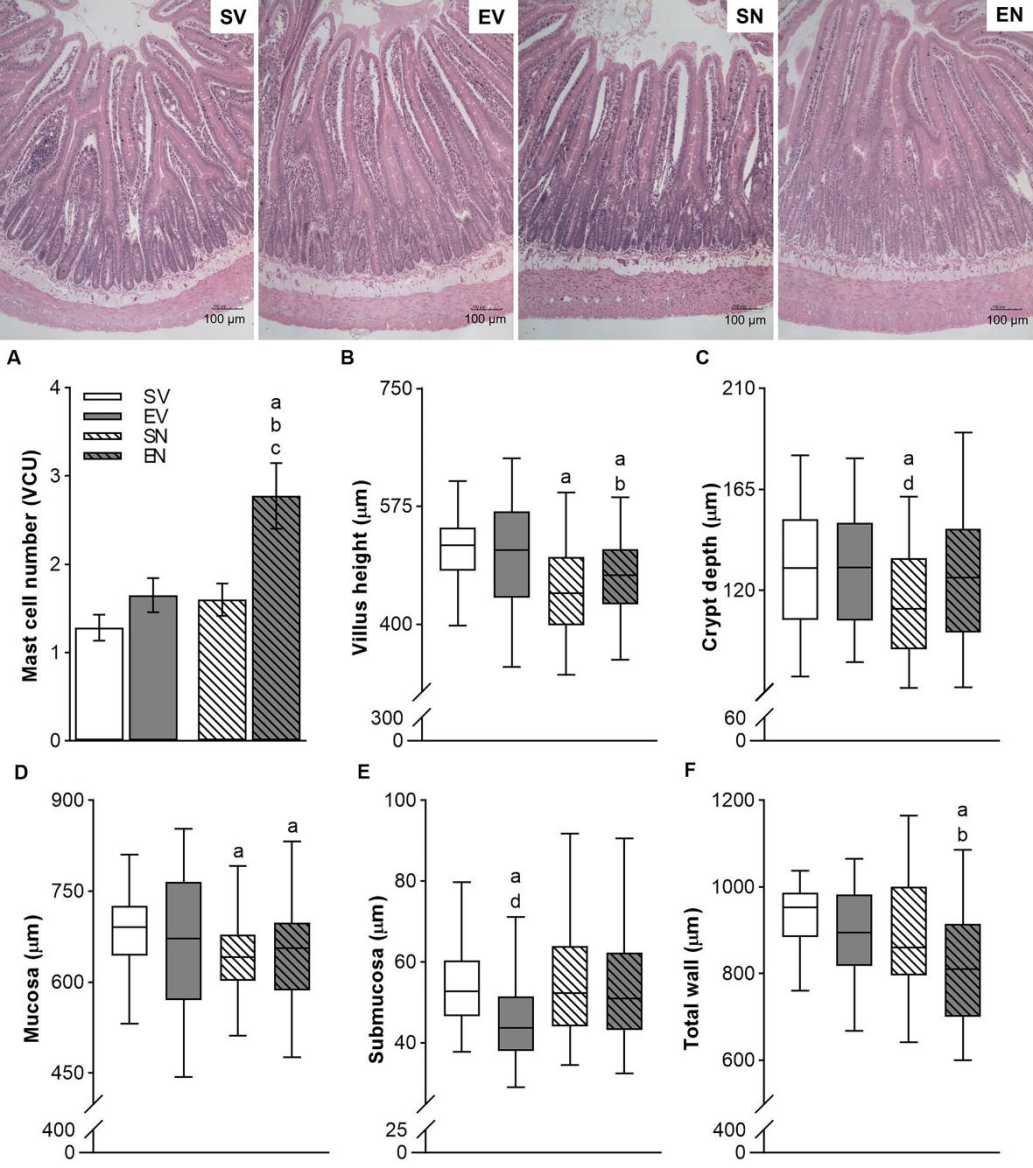
**Top**, Representative photomicrographs of the groups. Hematoxylin and eosin staining, 10× magnification. Scale bar, 100 µm. **A**, Quantification of intestinal mast cells and morphometric duodenal measurements of (**B**) villus height, (**C**) crypt depth, and thickness of the (**D**) mucosa, (**E**) submucosa, and (**F**) total wall of sedentary vehicle (SV), exercise vehicle (EV), sedentary 5 mg/kg nandrolone (SN), and exercise 5 mg/kg nandrolone (EN) rats after the 28-day protocol. Data are reported as means±SE or median and interquartile range (25-75%) from n=7 animals/group. ^a^P<0.010 *vs* SV; ^b^P<0.050 *vs* EV; ^c^P<0.01 *vs* SN; ^d^P<0.001 *vs* EN (Kruskal-Wallis test followed by Dunn's *post hoc* test and ANOVA followed by Tukey's *post hoc* test for mast cells only).

**Figure 6:**
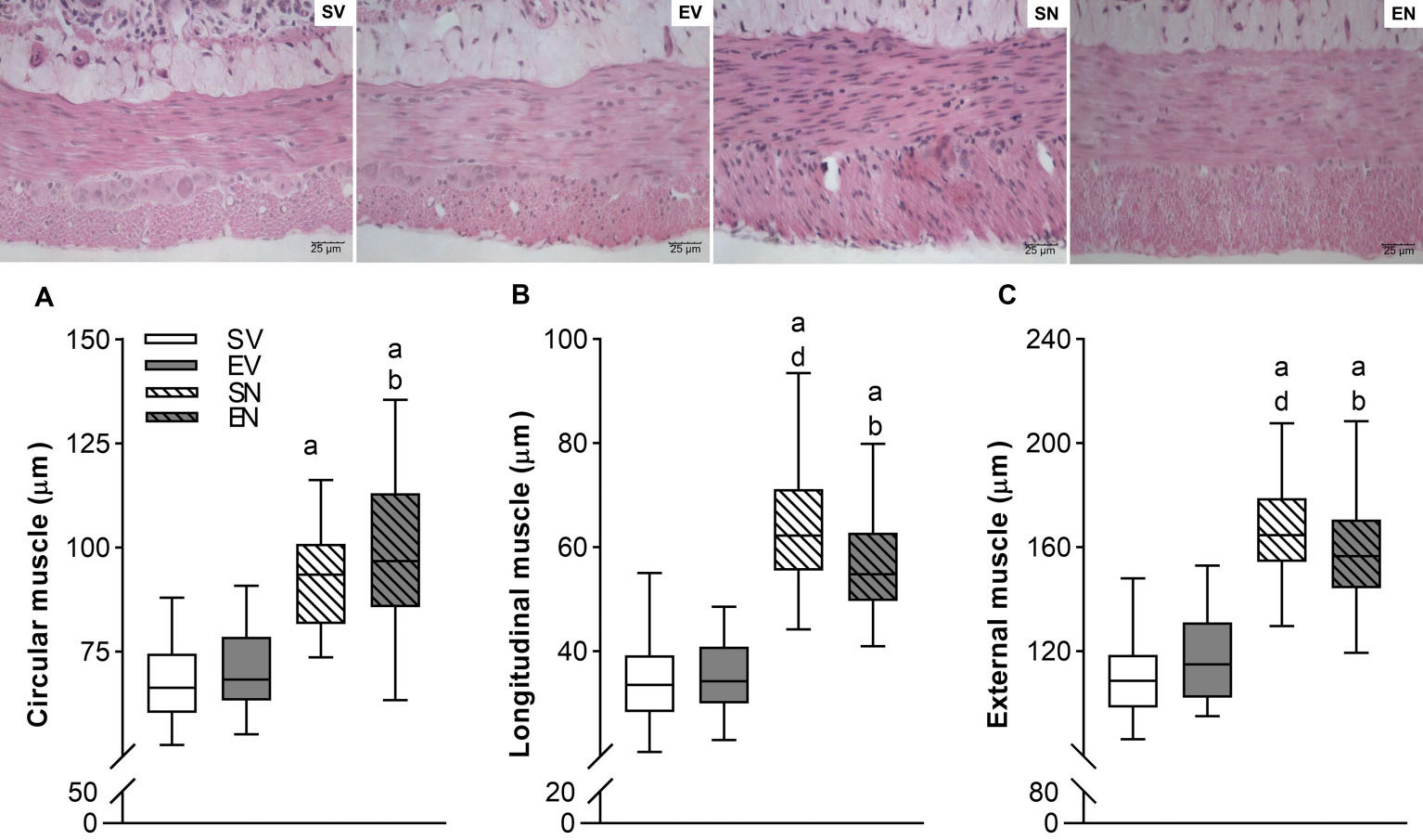
**Top**, Representative photomicrographs of the groups. Hematoxylin and eosin staining, 40× magnification. Scale bar, 25 µm. Morphometric duodenal thickness of the (**A**) circular, (**B**) longitudinal, and (**C**) external muscle layers of sedentary vehicle (SV), exercise vehicle (EV), sedentary 5 mg/kg nandrolone (SN), and exercise 5 mg/kg nandrolone (EN) rats after the 28-day protocol. Data are reported as median and interquartile range (25-75%) from n=7 animals/group. ^a^P<0.001 *vs* SV; ^b^P<0.001 *vs* EV; ^d^P<0.01 *vs* EN (Kruskal-Wallis test followed by Dunn's *post hoc* test).

Morphometric analysis showed that ND treatment decreased crypt depth, villus height, and mucosa thickness in the SN group compared with the SV group (Figure 5). There was also a thickening of the circular and longitudinal muscle layers of the duodenum compared with the respective mean values of the SV group (Figure 6). Moderate swimming workout decreased the submucosa thickness. The swimming protocol combined with ND treatment restored normal crypt depth but reduced total wall thickness (Figure 5). Compared with respective control values, the EN group exhibited an increase in the number of intestinal mast cells (2.60±0.46 cells/VCU *vs* 1.28±0.14 cells/VCU for SV group, Cohen's d=0.70; 1.65±0.19 cells/VCU for EV group, and 1.60±0.18 cells/VCU for SN group, Cohen's d=0.70; Figure 5A). Interestingly, there was a strong negative linear correlation between mean small intestine transit time and number of mucosal mast cells per villus/crypt in both the SV and EV groups (R=-0.93 and -0.89, respectively; P<0.05), being such negative linear correlation prevented by ND treatment.

### Assessment of body composition and blood lactate

Moderate-intensity swimming exercise increased blood lactate levels in both vehicle- and ND-treated rats (6.2±0.1 and 6.3±0.2 mmol/L, respectively, *vs* 4.7±0.2 mmol/L for SV group, Cohen's d=1.28, and 4.8±0.2 mmol/L for SN group, Cohen's d=1.15, respectively) (Table 1). Exercise combined with ND treatment induced a transient reduction of body water content, with decreases in TBW (141.5±2.3 mL), ECF (63.8±1.6 mL), and ICF (77.2±1.6 mL) compared with the SV group (160.4±4.4 mL, 69.9±2.5 mL, and 90.4±2.4 mL, respectively). The EV group also exhibited lower ICF values (78.8±3.3 mL), with a reduction of fat mass (75.3±5.0 g) and an increase of fat-free mass (219.2±6.1 g) compared to the SV animals (95.3±5.7 g and 196.3±7.6 g, respectively). BMI analysis showed that all trained and/or ND-treated groups exhibited a significant reduction of BMI over time (day 1 *vs* day 28). Moreover, both ND-treated groups (SN 3.3±0.6 g and EN 1.8±0.8 g) showed weight loss over the weeks in contrast to the natural weight gain in control animals (SV 4.6±0.3 g and EV 5.3±1.1) (Table 1).

**Table 1:** Body composition and blood lactate level in sedentary and exercised rats after the treatment with nandrolone decanoate or vehicle.

Parameter	Groups			
	SV	EV	SN	EN
Blood lactate (mmol/L)	4.7±0.2	6.2±0.1^a^	4.8±0.2	6.3±0.2^a,c^
Weight gain per week (g)	4.6±0.3	5.3±1.1	-3.3±0.6^a^	-1.8±0.8^a,b^
Body weight (g)				
Day 1	279.3±8.6	273.2±7.8	300.7±3.4	279.0±5.8
Day 14	291.7±10.4^#^	294.5±9.2	303.8±4.1	272.8±5.5
Day 28	297.8±12.4^#^	301.4±10.1*	289.2±6.0**	265.6±3.8
Total body water (mL)				
Day 1	136.4±2.4	134.2±5.9	143.2±4.1	138.6±4.3
Day 14	160.4±4.4	143.7±5.6	156.6±3.9	141.5±2.3^a^
Day 28	158.2±5.3	151.9±6.7	152.5±5.1	145.4±4.1
Intracellular fluid (mL)				
Day 1	73.4±0.9	71.2±4.5	79.1±3.0	77.9±2.4
Day 14	90.4±2.3	78.8±3.3^a^	85.5±2.4	77.6±1.6^a^
Day 28	86.2±2.4	79.2±5.4	79.8±2.8	78.9±3.5
Extracellular fluid (mL)				
Day 1	63.1±2.1	62.4±1.9	63.5±1.3	61.1±1.3
Day 14	69.9±2.5	64.8±2.3	71.0±1.7	63.8±1.6^a,c^
Day 28	71.9±3.0	72.7±2.2	72.2±3.0	66.3±1.9
Fat-free mass (g)				
Day 1	186.5±3.2	182.6±8.3	206.8±5.4	190.0±4.9
Day 14	196.3±7.6	219.2±6.1^a^	213.9±5.4	193.3±3.2^b^
Day 28	215.9±7.3	207.5±9.2	208.3±7.0	198.6±5.7
Fat mass (g)				
Day 1	92.8±6.5	90.5±4.3	93.8±4.6	88.9±5.7
Day 14	95.3±5.7	75.3±5.0^a^	89.9±4.7	79.5±3.7
Day 28	81.9±5.9	93.9±10.4	80.8±1.8	67.0±3.1^b^
Body mass index				
Day 1	10.6±0.3	10.6±0.2	10.6±0.1	10.5±0.2
Day 14	10.4±0.3	10.0±0.2	10.7±0.1	9.8±0.1
Day 28	10.3±0.3	9.2±0.2*	9.3±0.2**	9.0±0.4***

SV: sedentary vehicle; EV: exercise vehicle; SN: sedentary nandrolone; EN: exercise nandrolone. The data are reported as means±SE from n=7 animals/group. ^a^P<0.050 *vs* SV; ^b^P<0.050 *vs* EV; ^c^P<0.010 *vs* SN; ^#^P<0.01 *vs* SV on day 1; *P<0.05 *vs* EV on day 1; **P<0.010 *vs* SN on day 1; ***P<0.010 *vs* EN on day 1 (ANOVA followed by Tukey's *post hoc* test).

## Discussion

ND treatment had pleiotropic effects on gut morphofunctional properties in rats, which included accelerated gastric emptying, altered duodenal morphometry, and loss of the correlation between intestinal transit time and mucosal mast cells. The ACB technique allowed the noninvasive real-time evaluation of the *in vivo* gut motility profile in all experimental groups, showing accelerated GE rate of a solid test meal and a transiently slowed small intestine transit time. Moderate swimming workouts combined with ND treatment partially reversed these effects, although intestinal contractility and morphology remained impaired. ND treatment also disturbed GI smooth muscle contractility *in vitro* and increased the number of duodenal mast cells and smooth muscle layer thickness. These findings indicated that ND treatment had deleterious effects on the gastrointestinal tract, at least in the initial stages of misuse. Gut dysmotility may interfere with digestion and the absorption of nutrients and drugs, with putative negative consequences on energy balance and health (30).

Low-to-moderate-intensity exercise appears to protect the gut and improve its function, whereas the intensity of 65-80% of maximal aerobic capacity (VO_2_ max) may delay gastrointestinal motility (11-14). High-intensity swimming workouts delay the GE of liquids, decrease gastric contractility, and increase gastric distensibility. This phenomenon appears to be related to an acid/base imbalance caused by blood lactate accumulation during anaerobic exercise and can be reversed by sodium bicarbonate pretreatment (26). We have previously shown that extracellular acidosis due to strenuous exercise correlates with *in vitro* cholinergic inhibition of gastric fundus contractility via the Gq/11 protein-phospholipase C signaling cascade (27). Although the present study found that moderate-intensity exercise alone did not affect the GE of a solid test meal, it delayed the onset of GE acceleration elicited by ND treatment.

ND treatment and moderate exercise have opposite effects on small intestine transit rates. Physical exercise accelerated small intestinal transit in human studies (13,31). In contrast, our ND treatment alone transiently slowed the intestinal transit, but this change was normalized over time. González-Montelongo et al. (7) reported that testosterone altered the frequency peaks and increased the amplitude of ileal contractions in mice, leading to changes in intestinal transit. Gut motility is a highly modifiable event throughout intestinal segments. The proper regulation of intestinal transit time is essential for physiological absorptive processes, which also influence the mucosal immune system (15).

We observed an enhancement of CCh-mediated signaling through muscarinic receptors in the gastric tissue of sedentary ND-treated rats. Both the antrum and fundus exhibited a significantly higher magnitude of maximal tension after ND treatment compared with the SV group. Such improvements in cholinergic contractile reactivity may explain the acceleration of the GE rate. In addition, changes in circular and longitudinal smooth muscles have distinct effects on GI motility. Hypertrophy of the circular layer is closely related to hyperresponsiveness to contractile agents, while the longitudinal muscle exhibits greater sensitivity to relaxing agents (32). Chronic aerobic swimming workouts changed GI contractile reactivity by reducing tissue responsiveness to KCl and CCh, decreasing circular muscle thickness, and increasing the longitudinal muscle in the rat ileum (33).

Although moderate exercise alone did not have a notable impact on the *in vitro* contractile profile of GI segments, it reversed the increase in CCh-mediated gastric reactivity in the ND-treated group. Duodenal contractions were intensely stimulated in ND-treated rats exposed to swimming workouts, possibly due to a higher tissue responsiveness to CCh and a decrease in the SNP relaxant pathway. Notwithstanding, occasional changes in one segment of the small intestine can be compensated along the tube through its different contraction patterns (30). Hence, changes in duodenal contractility did not alter the intestinal transit rate *in vivo* but could have contributed to the acceleration of GE.

Some effects of supratherapeutic doses of ND are related to the enzymatic aromatization of estradiol in peripheral tissues, which interacts with estrogen receptors (2). The effects of ND and endogenous sex hormones on the GI tract smooth muscle are still uncertain. Androgenic receptors expressed in the GI smooth muscle (34,35) serve as a potential target for direct actions of AAS. Increased serum estrogen levels induce gastric hypercontractility (9) while experimental estradiol deficiency delays GE by activation of the nitric oxide inhibitory pathway (36). Considering this, we hypothesized that high levels of estradiol generated from the conversion of nandrolone may also contribute to the present GE acceleration.

Mast cell activation and release of mediators have been shown to play an important role in GI permeability, secretion, and motility (16). *In vivo* and *in vitro* studies suggest that sex hormones regulate the function and distribution of mast cells in various tissues and mediate different immune responses (37,38). The treatment of female rats with estradiol, progesterone, testosterone, and dihydrotestosterone favored the release of histamine by peritoneal mast cells (37). Our study showed that the combination of ND treatment and swimming workouts increased the number of duodenal mast cells in rats. Based on these results, one should consider that mucosal mast cell hyperplasia does not necessarily translate into an increase in the activation or degranulation of these cells (38).

Although the activity of mast cells is linked to intestinal permeability and absorption, the relationship between these cells and gut motility is still uncertain (15). We found a strong negative correlation between the number of mast cells and GI transit in both the sedentary and trained control groups, indicating that a lower number of mast cells in the duodenal mucosa favored the prolonged transit of food debris in the small intestine. ND treatment did not correlate with mast cell number and intestinal transit, although moderate physical exercise increased the number of mast cells. Understanding the mechanisms by which AAS and physical workouts influence GI motility and local immunity may have a major impact on physiological function, pharmacological therapy, and clinical practice.

The recent growth of AAS misuse and abuse, mainly among non-athletes, is worrisome for health, especially considering that the putative side effects of AAS remain uncertain (4). Animal models of physical stress are suitable for understanding the physiological adaptations that occur in response to exercise following AAS treatment, diets, and diseases (17,22,26), since clinical studies are limited by ethical concerns (11,12). Blood lactate threshold is considered the gold standard for determining the intensity of swimming workouts in rats (21,39). Brito et al. (22) reported that swimming sessions with a 3-6% overload elevated blood lactate levels, characterizing these sessions as moderate intensity. The training protocol used in this study stimulated aerobic gains, corroborating previous reports in which rats subjected to swimming with a 5% body weight overload exhibited increased lactate concentrations without exceeding the anaerobic threshold (39,40). Furthermore, with a similar model of endurance training, de Araújo et al. (20) showed no significant repercussions in antioxidant activity and corticosterone levels after 4 weeks of swimming in rats.

In conclusion, our data revealed that ND supratherapeutic use accelerated gastric emptying over time through enhancement of CCh-mediated reactivity in the gastric tissue strips, especially in sedentary animals. ND also changed duodenal morphometry and abolished the correlation between intestinal transit and mucosal mast cells. Combined with moderate swimming, the harmful effects of the drug were partially compensated, although intestinal contractility and morphology remained impaired.
